# Single-cell transcriptomics uncovers persistent hepatic immunometabolic remodeling following SARS-CoV-2 infection in obese hosts

**DOI:** 10.3389/fimmu.2026.1910050

**Published:** 2026-10-14

**Authors:** Wei Qian, Xiaoqin Wei, Soo-Jeung Park, Josefina Garcia Diaz, Tina Comlekoglu, Jane Yu, Jie Sun, Young S. Hahn

**Affiliations:** 1 Beirne B. Carter Center for Immunology Research, University of Virginia, Charlottesville, VA, United States; 2 Department of Microbiology, Immunology and Cancer Biology, University of Virginia, Charlottesville, VA, United States; 3 Division of Infectious Diseases and International Health, Department of Medicine, University of Virginia, Charlottesville, VA, United States

**Keywords:** liver, macrophages, obesity, SARS-CoV-2, scRNA-seq

## Abstract

SARS-CoV-2 infection affects multiple organs, and chronic post-viral tissue injury is often associated with persistent immune dysregulation. Obesity and related metabolic disorders are major risk factors for severe COVID-19 and post-acute sequelae of SARS-CoV-2 infection (PASC), including liver dysfunction. To define hepatic responses during post-acute infection, we studied lean and diet-induced obese mice at 7 and 28 days following infection with mouse-adapted SARS-CoV-2. Compared with lean mice, obese mice exhibited persistent lung inflammation and fibrosis, along with liver injury characterized by increased lipid accumulation, cellular apoptosis, and metabolic dysfunction. Single-cell RNA sequencing revealed obesity-associated alterations in hepatic immune and metabolic programs during post-acute infection. In particular, hepatic macrophages in obese mice showed sustained inflammatory and metabolic transcriptional alterations, while hepatocytes displayed altered programs associated with gluconeogenesis and lipid metabolism. Endothelial cells also exhibited persistent transcriptional changes linked to immune activation and prothrombotic pathways. These alterations remained evident at 28 days post-infection, suggesting incomplete restoration of immune and metabolic homeostasis in obese liver. Collectively, our findings reveal persistent obesity-associated hepatic immunometabolic remodeling following SARS-CoV-2 infection and identify cellular and molecular programs that may contribute to post-acute liver sequelae in metabolically compromised hosts.

## Introduction

The COVID-19 pandemic has posed an unprecedented global health challenge, causing significant morbidity and mortality worldwide. Although initially characterized as a respiratory illness, COVID-19 is now recognized as a systemic disease affecting multiple organ systems, including the cardiovascular, renal, neurological, and hepatic compartments (1, 2). In parallel, obesity and associated metabolic disorders represent a growing global health burden, with metabolic syndrome estimated to affect more than 1.5 billion adults worldwide (3). Obesity is a major risk factor for severe acute COVID-19 and has also been associated with an increased risk of post-acute sequelae of SARS-CoV-2 infection (PASC) (4, 5). However, how pre-exiting metabolic dysfunction influences tissue responses and recovery following infection remains incompletely understood.

Liver injury is a common extrapulmonary manifestation of COVID-19 and has been reported in a substantial proportion of hospitalized patients (6). The underlying pathophysiology is likely multifactorial, involving systemic inflammation, metabolic stress, hypoxia, drug-induced injury, and potentially direct effects of SARS-CoV-2 infection on susceptible cells (7). SARS-CoV-2 proteins, including Nsp1, can interfere with host antiviral immune responses (8, 9), but whether viral factors or viral persistence contribute to sustained hepatic alterations after acute infection remains unclear. Although liver abnormalities are transient in many patients, persistent alterations in liver enzymes and other hepatic biomarkers have been reported after acute infection (10), suggesting hepatic involvement in PASC​. Individuals with obesity or type 2 diabetes may be particularly susceptible to COVID-19-associated liver complications (11), as pre-existing metabolic dysfunction is associated with chronic low-grade inflammation and altered tissue immune responses to SARS-CoV-2 infection (12). Consistently, preclinical models of obesity show increased disease severity following SARS-CoV-2 infection, including greater morbidity, pulmonary pathology, and mortality​ (13–17). Importantly, obesity is also associated with impaired recovery after acute infection, characterized by delayed resolution of inflammation and persistent metabolic and tissue abnormalities (15–17), suggesting that pre-existing metabolic dysfunction may not only amplify acute disease but also alter the trajectory of tissue recovery following infection. Despite these advances, how obesity alters the cellular and molecular responses of the liver to SARS-CoV-2 infection over time remains poorly understood. In particular, the temporal dynamics of liver immune responses and tissue recovery in obese versus lean hosts have not been systematically characterized at single-cell resolution.

To address this gap, we infected lean and diet-induced obesity (DIO) mice with SARS-CoV-2 and performed single-cell RNA sequencing (scRNA-seq) of liver tissue at 0, 7, and 28 days post-infection (d.p.i.). These time points enabled us to examine hepatic immune responses and cellular remodeling states across acute infection and post-acute recovery. We found that obese mice exhibited greater liver injury and myeloid inflammation during acute infection, while inflammatory and metabolic alterations across macrophages, hepatocytes, and endothelial cells remained evident at 28 d.p.i. These findings together reveal persistent hepatic immunometabolic remodeling following SARS-CoV-2 infection in obese hosts and provide a cellular framework for understanding impaired liver recovery after infection.

## Materials and methods

### Mice

Male DIO C57BL/6J mice (with 60% kcal fat chow over 20 weeks) (strain no. 380050) and age-matched control male mice (strain no. 380056) were purchased from Jackson Laboratory at 24 weeks of age and housed for at least 2 weeks with 60% kcal fat chow (Research Diets, #D12492) or normal chow before and throughout the experimental period. The mice were housed under specific pathogen-free conditions at University of Virginia (UVA). Only male mice were used in this study.

### Viral infection in lean and obese mice

The SARS-CoV-2 mouse-adapted strain MA10 virus was stored in the lab (18, 19). For SARS-CoV-2 MA10 infection, the mice were anesthetized via IP injection of an anesthetic mixture containing ketamine (100 mg/kg) and xylazine (10 mg/kg) and subsequently infected intranasally with 10^4^ plaque-forming units (PFU) MA10 virus in 50 μL of DMEM, while the control mice were given 50 μL of DMEM. The mortality rate of mice determined either by being found dead in the cage or by being euthanized after reaching 70% of their initial body weight in accordance with the humane endpoint. At the designated endpoint, the mice were humanely euthanized with a ketamine–xylazine overdose (200 mg/kg ketamine and 20 mg/kg xylazine), followed by cervical dislocation. The animal experiments were approved by UVA Institutional Animal Care and Use Committee (protocol no. 4370). All work with SARS-CoV-2 infections were approved under Animal Biosafety Level 3 (ABSL3) conditions and were performed with approved standard operating procedures and safety conditions from UVA Institutional Review Board (IRB 13166).

### Tissue collection and immunostaining

Mice were randomly selected for tissue collection at 0, 7, and 28 d.p.i. To distinguish circulating from tissue-localized leukocytes, the mice were intravenously labeled with 2 μg anti-CD45 antibody for 5 min before sacrifice (20). Blood was collected for serum preparation, and bronchoalveolar lavage (BAL) fluid was collected for viral titration. Lung and liver tissues were harvested for flow cytometry and scRNA-Seq or fixed in 10% formalin for histological analysis. Fixed samples were embedded in paraffin and cut into 5-um sections for H&E and Masson’s trichrome staining by the Research Histology Core at UVA. The slides were digitalized at 20× resolution by the Biorepository and Tissue Research Facility at UVA. Lung inflammatory areas were blindly quantified using QuPath (21).

For immunofluorescence staining, paraffin-embedded lung sections were deparaffinized in xylene, were rehydrated through graded ethanol solutions, and underwent heat-induced antigen retrieval using 1× Agilent Dako Target Retrieval Solution (pH 9, S236784) for 20 min. After the PBS washes, the sections were permeabilized with 0.5% Triton X-100 and 0.05% Tween-20 in PBS for 1 h at room temperature (RT) and then incubated overnight at 4 °C with rabbit anti-collagen I (1:100, Abcam, ab270993) and mouse anti-alpha smooth muscle actin (1:100, Abcam, ab7817) diluted in Dako Antibody Diluent. The sections were then incubated for 2 h at RT with goat anti-rabbit IgG Alexa Fluor 488 (1:300, ThermoFisher, A-11008) and goat anti-mouse IgG AF555 (1:300, ThermoFisher, A-21422). Nuclei were counterstained with DAPI (1:5,000) for 3 min, and the sections were mounted with ProLong Diamond Antifade Mountant. Images were acquired using a ×20 objective on an Olympus BX63 fluorescence microscope to quantify Collagen I^+^ and α-SMA^+^ areas. Image analysis was performed using OlyVIA and/or QuPath software.

Hepatic apoptosis was assessed by TUNEL staining using a TUNEL assay kit (Abcam, ab206386) according to the manufacturer’s instructions. The stained sections were examined using an Olympus BX40F4 microscope (Southern Microscope), and TUNEL^+^ areas were quantified using QuPath.

### Tissue processing and flow cytometry

Livers were minced and transferred into 20 mL EGTA buffer, followed by incubation at 37°C for 10 min with gentle agitation. Samples were centrifuged at 2,500 rpm for 5 min at 4°C, and the pellets were resuspended in digestion buffer containing collagenase IV and incubated at 37 °C for 30 min with agitation. The cell suspensions were filtered, washed with HBSS, and centrifuged at 2,500 rpm for 5 min at 4 °C. The red blood cells were lysed using RBC lysis buffer for 1 min at RT. The cell pellets were resuspended in 40% Percoll (Sigma) and centrifuged at 2,500 rpm for 10 min at 4 °C. Following the removal of the upper layer, the cells were washed, centrifuged at 3,000 rpm for 10 min at 4 °C, washed once with FACS buffer, and centrifuged at 3,000 rpm for 5 min. The final cell pellets were resuspended in FACS buffer for downstream analysis.

For flow cytometric analysis, liver single-cell suspensions were stained with Zombie NIR viability dye (BioLegend, 423106) and incubated with Fc receptor blocker for 10 min at RT, followed by surface staining with antibodies for 30 min on ice. The antibodies used in this study are listed in **Supplementary Table S1**. Samples were acquired on an Attune NxT flow cytometer (Invitrogen) and analyzed using FlowJo software. The gating strategy used to identify hepatic immune cells is shown in **Supplementary Figure S2**. Each biological replicate represents an individual mouse.

### Liver functional tests

Blood glucose levels were measured using a colorimetric glucose assay kit (Abcam, ab65333), and cholesterol levels were measured using a colorimetric cholesterol assay kit (Abcam, ab65390) according to the manufacturer’s instructions. Serum alanine aminotransferase (ALT) levels were assessed using the Mouse ALT SimpleStep ELISA kit (Abcam, ab282882) according to the manufacturer’s instructions.

### Virus titer measurement

Viral titers in BAL fluid were determined by plaque assay using Vero E6 cells seeded 1 day prior to infection (18). In brief, BAL samples were serially diluted 10-fold and added to cell monolayers and incubated at 37 °C and 5% CO_2_ for 1 h, with gentle agitation every 15 min. After adsorption, the inoculum was removed, and the cells were washed twice with pre-warmed PBS before being overlaid with a 1:1 mixture of 2.4% Avicel and 2× DMEM supplemented with 2% FBS. At 3 days after infection, the cells were fixed with 4% paraformaldehyde and stained with 0.1% crystal violet. The plaques were counted, and viral titers were calculated as PFU/mL of BAL fluid.

### scRNA-Seq analysis

Liver single-cell suspensions isolated from lean and obese mice at 0, 7, and 28 d.p.i. were processed for single-cell gene expression library construction with Chromium Single Cell 3′ Reagent Kit (10x Genomics) according to the manufacturer’s protocol. For each experimental condition, cells isolated from three individual mice were pooled before library preparation. Sequencing reads were aligned to the mouse reference genome and quantified using the 10× Genomics Cell Ranger pipeline. Putative doublets were identified and excluded using the *scDblFinder* R package. Quality control and downstream analyses were performed using *Seurat* (v4.1.1). Cells with >200 detected genes, >1,000 unique molecular identifiers (UMIs), and <5% mitochondrial gene content were ensured for further analysis. Following quality control, gene expression data were normalized and subjected to dimensionality reduction and unsupervised clustering. Cell clusters were annotated based on the expression of established lineage markers, with reference to published marker genes and the CellMarker database. For detailed analyses of hepatic macrophages and T cells, the corresponding compartments were subsetted and reclustered based on their transcriptional profiles.

Differential gene expression (DEG) analysis was performed in *Seurat* to identify transcriptional differences between experimental conditions and cell populations. Gene set enrichment analysis (GSEA) was performed using the *clusterProfiler* R package based on marker genes identified using the *FindAllMarkers* function. Gene Ontology and pathway enrichment analyses were used to characterize biological processes associated with different transcriptional states. Gene signature scores were calculated at the single-cell level to assess the relative enrichment of selected inflammatory, metabolic, and cellular programs across experimental conditions and are presented descriptively unless specific statistical comparisons are indicated.

Cell–cell communication was analyzed using *CellChat* to infer potential ligand–receptor interactions among hepatocytes and myeloid populations. Interaction networks were compared between lean and obese mice at 7 and 28 d.p.i. The results were interpreted as predicted intercellular communications based on gene expression patterns rather than direct measurements of functional cell–cell signaling.

### Statistical analysis

Statistical analyses were performed using GraphPad Prism version 9. For comparisons between two independent groups, two-tailed Mann–Whitney tests were used. Two-way ANOVA followed by appreciated multiple-comparison tests was used for experiments involving multiple groups and/or time points. Survival curves were compared using the log-rank Mantel–Cox test. Data are presented as mean ± SEM, with each data point representing an individual mouse unless otherwise indicated. Sample size (*n*), statistical tests, and significance levels for individual experiments are specified in the corresponding figure legends.

## Results

### Obesity exacerbates morbidity and persistent lung pathology following SARS-CoV-2 infection ​

To determine the pathological impact of obesity on the outcome of SARS-CoV-2 infection, we infected lean and obese mice with mouse-adapted SARS-CoV-2 strain MA10 and evaluated disease progression and tissue pathology at 0, 7, and 28 d.p.i. Upon infection, obese mice exhibited reduced survival and sustained clinical disease compared with lean mice (**Figures 1A–C**). A histological examination of lung tissue revealed prominent inflammatory cell infiltration during acute infection that remained evident at recovery stage (**Figure 1D**). Masson’s trichrome staining further showed increased collagen deposition in the lungs of obese mice, with more extensive fibrotic remodeling seen at 28 d.p.i. (**Figure 1E**). Consistent with this finding, at 28 d.p.i., obese mice exhibited an increased expression of the fibrotic markers α-SMA and collagen I (**Figures 1F, G**). Despite these pathological differences, viral loads were comparable between groups at day 7 and undetectable by day 28 (**Figure 1H**). Both groups had increased lung inflammation after infection, with a larger inflamed area observed in obese mice at 28 d.p.i., although the difference did not reach statistical significance (**Supplementary Figures S1A, B**). Collectively, these findings suggest that obesity exacerbates SARS-CoV-2-associated morbidity and promotes persistent lung pathology despite comparable viral clearance.

**Figure 1:**
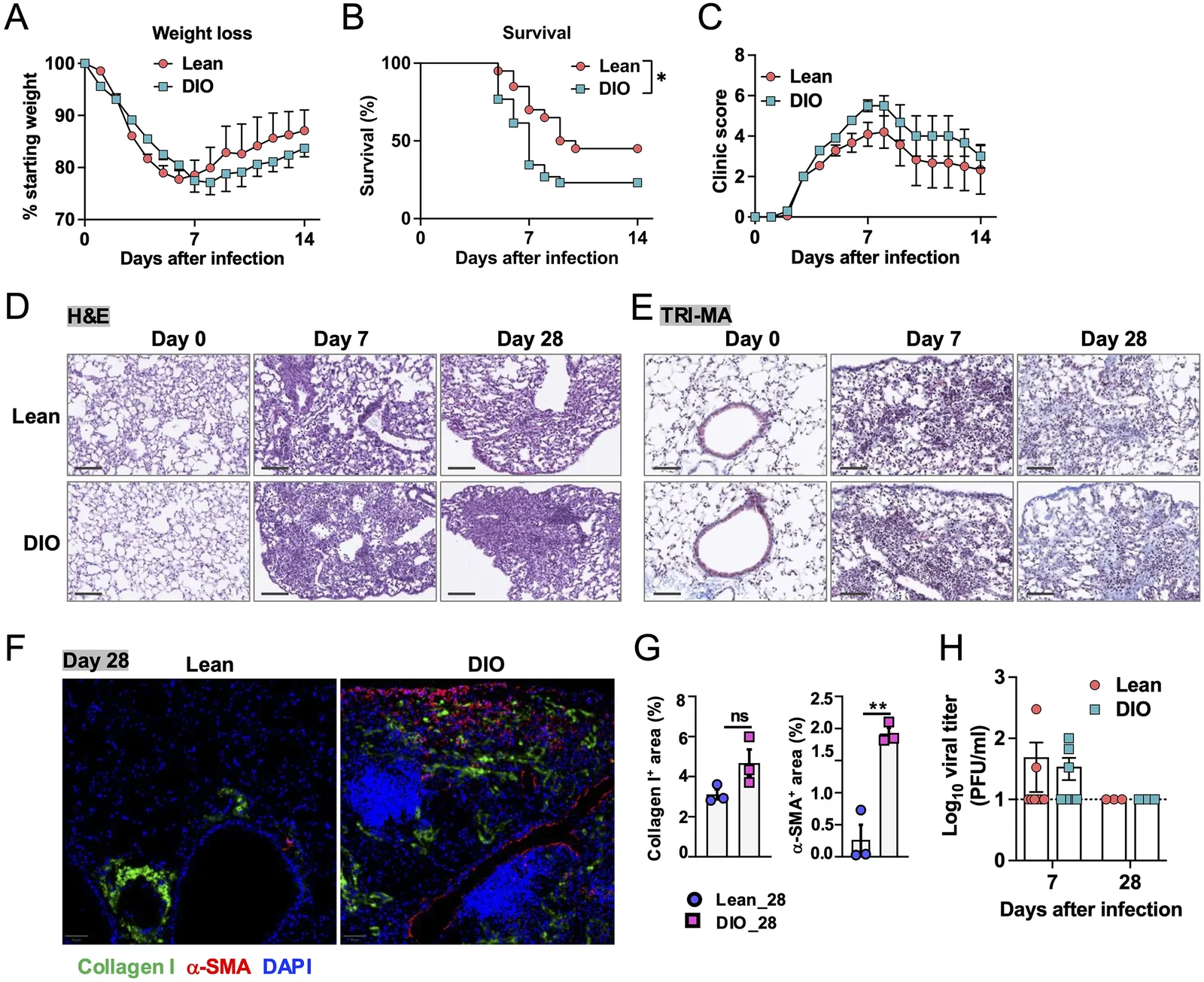
Increased morbidity of obese mice following SARS-CoV-2 infection​. **(A–C)** DIO and lean mice were infected with SARS-CoV-2 MA10 (10^4^ pfu/mouse) and monitored for body weight change **(A)**, survival **(B)**, and clinical scores **(C)** for 2 weeks. Body weight data represent two independent experiments (lean, *n* = 10; DIO, *n* = 9), survival data represent four independent experiments (lean, *n* = 20; DIO, *n* = 25), and clinical scores represent three independent experiments (lean, *n* = 15; DIO, *n* = 14). **(D, E)** Representative lung histopathology from obese and lean mice at 0, 7, and 28 d.p.i. **(D)** H&E staining highlights inflammatory infiltration. **(E)** Masson’s trichrome (TRI-MA) staining reveals fibrotic collagen deposition. Scale bar, 100 μm. **(F, G)** Representative immunofluorescence images of lung sections at 28 d.p.i. stained for collagen I and α-SMA **(F)**, with quantification shown on the right **(G)**. Each symbol represents one mouse, *n* = 3 mice/group. **(H)** Viral titers in BAL fluid were measured by plaque assay using Vero E6 cells. Dots represent individual mouse, and the dashed line indicates the limit of detection. Regarding data in **(D–H)**, samples at 0 and 7 d.p.i. were pooled from two independent experiments, whereas samples at 28 d.p.i. were obtained from mice that survived to the endpoint. Data are represented as mean ± SEM unless otherwise indicated. Statistical significance was assessed by using log-rank Mantel–Cox test **(B)** and Mann–Whitney test **(G)**. ns, *p* > 0.05; *p < 0.05; **p < 0.01.

### Obesity enhances liver injury and inflammation following SARS-CoV-2 infection

Given that patients hospitalized with COVID-19 frequently exhibit hepatic dysfunction (22), we next assessed liver pathology and metabolic changes of the infected mice. Obese mice showed greater lipid droplet accumulation than lean mice at baseline, which was further increased during acute infection and remained elevated at 28 d.p.i., indicating virus-induced metabolic shift (**Figure 2A**). TUNEL staining showed that hepatic apoptosis was increased in obese mice and remained prominent at recovery (**Figure 2B**). We next assessed liver function by measuring hepatocyte damage and metabolic condition (23). There was increased glucose and cholesterol levels in obese mice up to 28 d.p.i. compared to infected lean mice (**Figure 2C**). Although these changes could be a direct result of obesity but not from viral infection, the ALT level was elevated in infected obese mice at 7 d.p.i. (**Figure 2C**), indicating that significant hepatocellular injury was most evident during acute infection.

**Figure 2:**
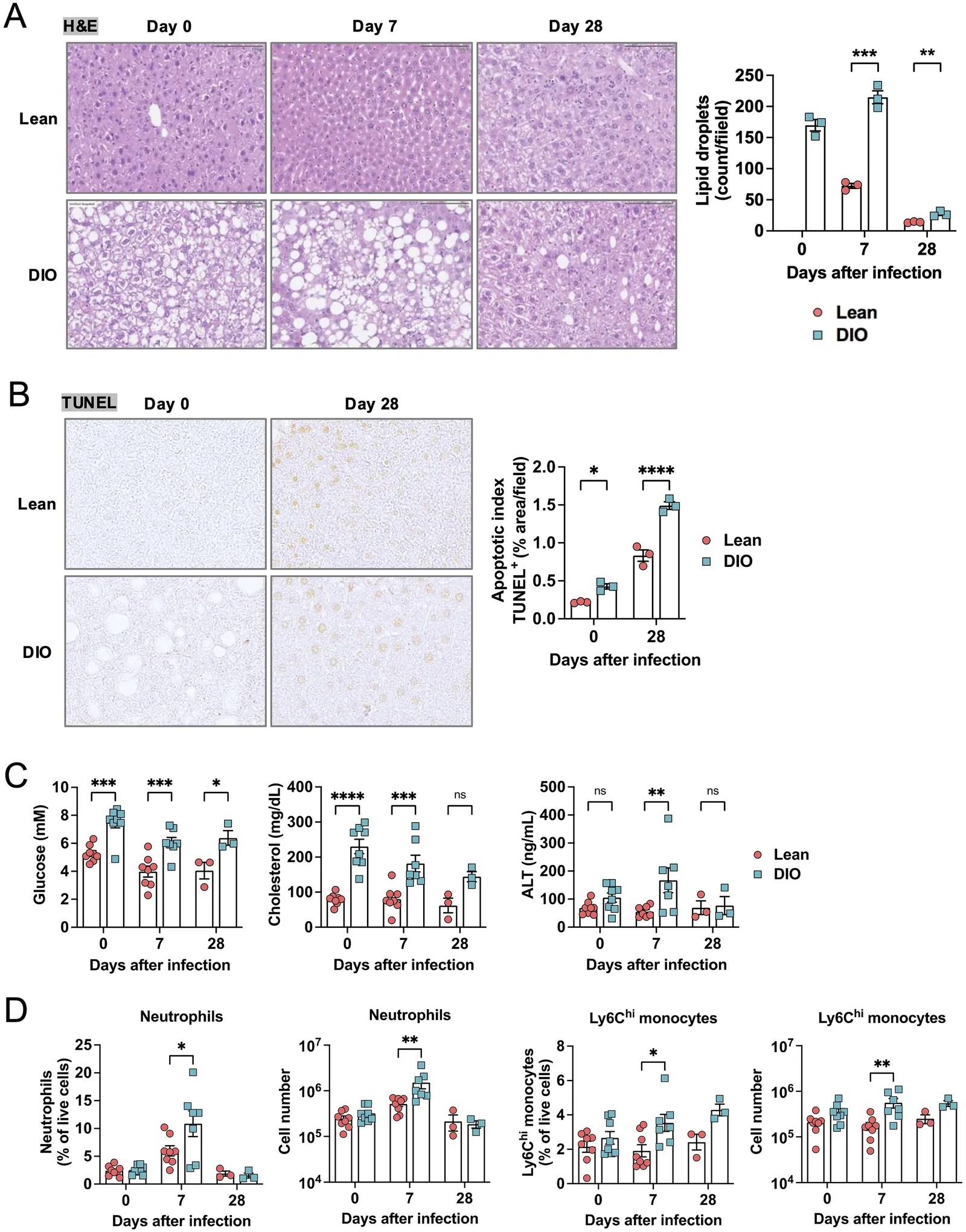
Obese mice exhibit liver inflammation and metabolic impairment after SARS-CoV-2 infection​. **(A)** Representative H&E staining of liver sections from obese and lean mice at 0, 7, and 28 d.p.i. and quantification of lipid droplets. Quantification is from one experiment, with each point representing an individual mouse. **(B)** Representative TUNEL staining of liver sections from lean and obese mice at 0 and 28 d.p.i. and quantification of the apoptotic index. Quantification is from one experiment, with each point representing an individual mouse. **(C)** Liver functional tests assessing for blood glucose, cholesterol, and ALT levels in lean and obese mice at 0, 7, and 28 d.p.i. **(D)** Frequency and absolute number of neutrophils and Ly6C^hi^ monocytes in liver parenchyma (i.v.CD45^-^) of obese and lean mice at 0, 7, and 28 d.p.i. Data are presented as mean ± SEM. Statistical significance was assessed by using Mann–Whitney test **(A, B)** and two-way ANOVA **(C, D)**. ns, *p* > 0.05; *p < 0.05; **p < 0.01; ***p < 0.001; ****p < 0.0001.

To determine the potential contribution of immune cells to liver damage, we performed flow cytometry on liver non-parenchymal cells. Notably, Ly6C^hi^ monocytes and neutrophils were increased in obese mice at 7 d.p.i. but declined thereafter (**Figure 2D**; **Supplementary Figure S2A**). Additional changes in macrophages, T cells, NK cells, and B cells were observed at baseline or during acute infection (**Supplementary Figures S2B**, **S3A**), while circulating neutrophils or monocytes showed limited differences between lean and obese groups (**Supplementary Figure S3B**). This suggests that the increase in hepatic immune cells in obese mice is largely due to enhanced tissue infiltration rather than increased circulating levels within the liver vasculature. Thus, obesity is associated with enhanced hepatic metabolic dysfunction and inflammation during acute SARS-CoV-2 infection, with lipid accumulation and apoptosis persisting after the acute inflammatory response subsides.

### scRNA-seq reveals hepatic cellular and transcriptional alterations after SARS-CoV-2 infection

To define the cellular programs underlying these hepatic abnormalities, we performed scRNA-seq on liver tissues from lean and obese mice at 0, 7, and 28 d.p.i. Major hepatic immune, stromal, and parenchymal populations were identified based on canonical lineage markers (**Figures 3A, B**), and the relative representation changed substantially across infection and recovery (**Figures 3C, D**). In lean mice, B and T cells progressively increased in relative abundance from day 7 to 28, whereas in obese mice this increase primarily occurred at day 28. Myeloid cells showed distinct temporal patterns, with neutrophils markedly increased at day 7 in both groups and more abundant in obese mice during acute infection. In addition, the proportion of hepatocytes decreased following infection in both mice. These findings reveal dynamic remodeling of the hepatic cellular landscape during infection and recovery, with distinct trajectories between lean and obese hosts.

**Figure 3:**
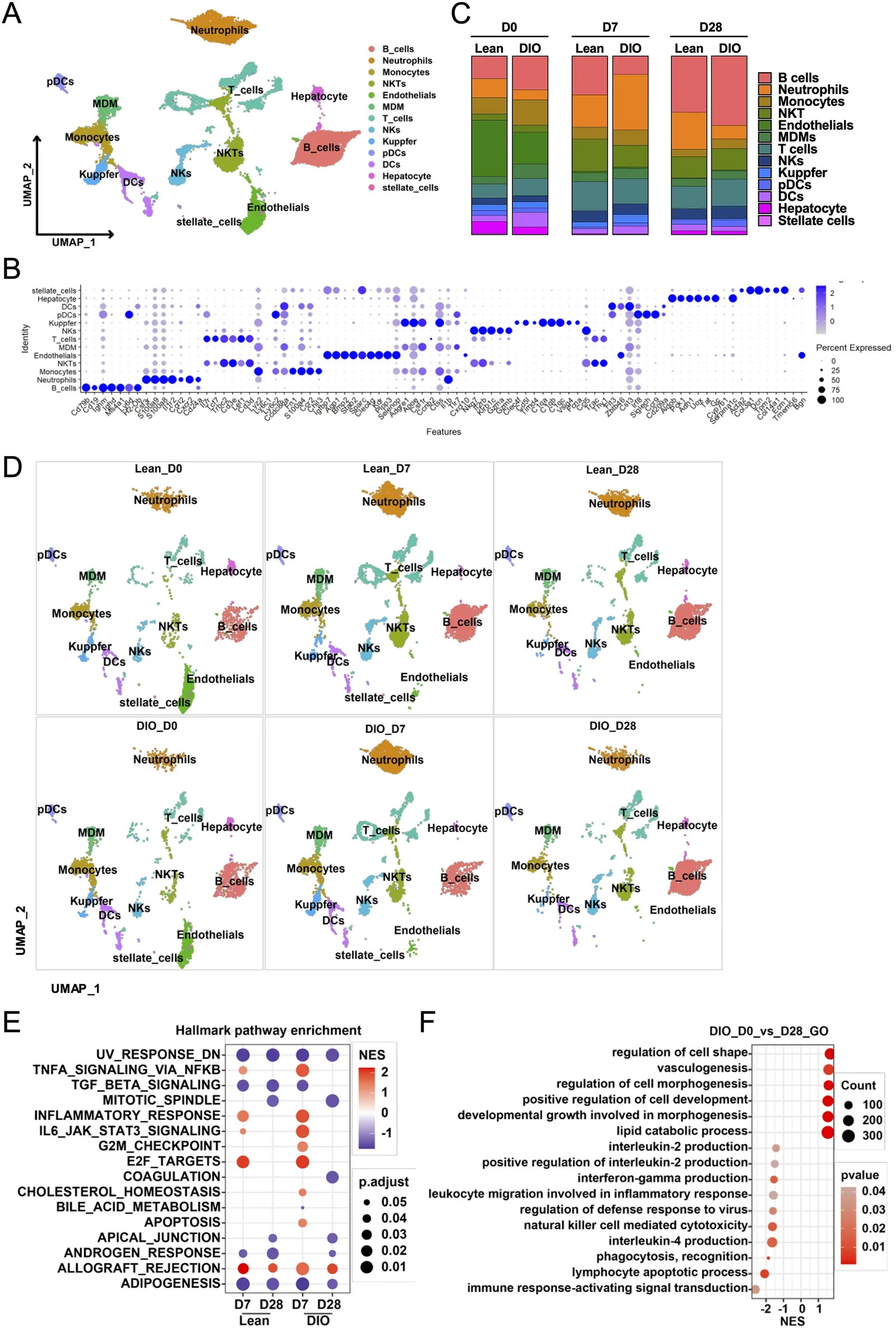
scRNA-seq reveals hepatic cellular and transcriptional alterations after SARS-CoV-2 infection. Liver single-cell suspensions from lean and obese mice at 0, 7, and 28 d.p.i were subjected to scRNA-seq. For each group, cells isolated from three individual mice were pooled before library preparation, resulting in one pooled scRNA-seq library per condition. **(A)** UMAP plots show global liver cell populations from six experimental groups. **(B)** Dot plot showing the expression of typical marker genes used for cell type annotation. **(C)** Relative representation of major clusters across the six experimental groups. **(D)** UMAP distribution of cell clusters stratified by group. **(E)** Hallmark pathway enrichment analysis comparing 7 or 28 d.p.i. with the corresponding baseline within lean and obese groups. **(F)** Gene Ontology enrichment analysis of DEGs in obese liver between D0 and D28.

These cellular alterations were accompanied by different transcriptional responses. At 7 d.p.i., obese livers showed enrichment of inflammatory, proliferative, and metabolic programs relative to lean livers, including IL6-JAK-STAT3 signaling, G2/M checkpoint, cholesterol homeostasis, and apoptosis (**Figure 3E**). Although several acute response programs subsided during recovery, obese livers at day 28 remained transcriptionally distinct from baseline, with enrichment of pathways related to cytokine production, leukocyte migration, NK-cell-mediated cytotoxicity, phagocytosis, and immune activation and reduced pathways associated with vasculogenesis, cell morphogenesis, and lipid catabolic processes (**Figure 3F**). The persistence of immune activation and diminished tissue structural metabolic signatures indicate prolonged liver inflammation and incomplete restoration of tissue homeostasis in obese mice after acute SARS-CoV-2 infection.

### Hepatic endothelial cells exhibit persistent transcriptional changes in obese mice

Endothelial cells integrate vascular, immune, and metabolic signals and may therefore be particularly sensitive to the inflammatory and metabolic environment associated with obesity (24). We observed that, overall, DEGs were greatly increased at 28 d.p.i. in both lean and obese mice, indicating that prior SARS-CoV-2 infection persistently altered endothelial cell transcription (**Figure 4A**). At 7 d.p.i., lean and obese groups exhibited distinct endothelial responses. Endothelial cells from obese mice preferentially expressed immune-associated signatures, including TGF-β signaling, T cell differentiation, neutrophil chemotaxis, type I interferon response, and antiviral defense, whereas endothelial cells from lean mice showed upregulation of pathways related to glucose homeostasis, phospholipid catabolic process, and carboxylic acid process (**Figure 4B**).

**Figure 4:**
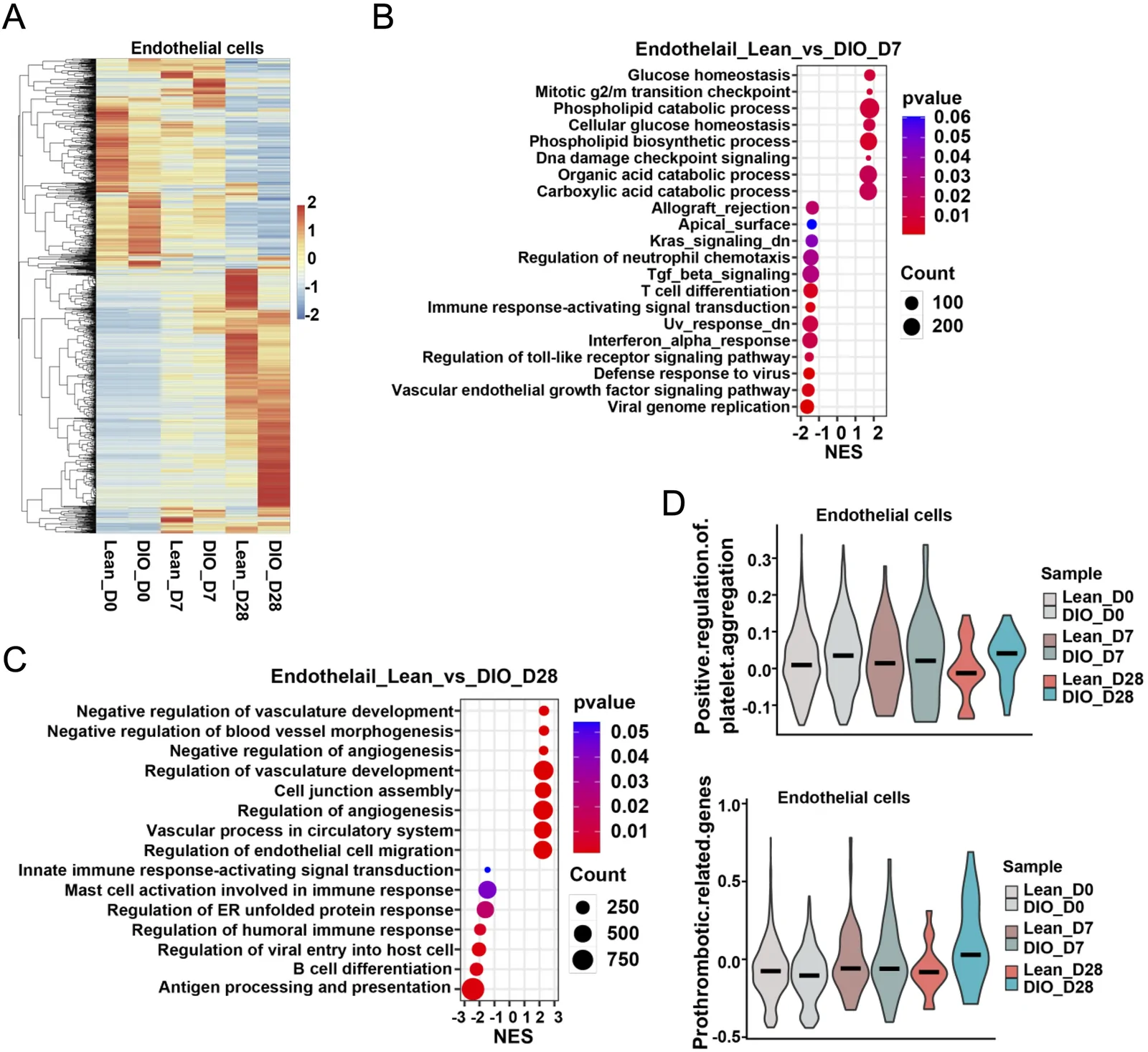
Hepatic endothelial cells exhibit persistent transcriptional changes in obese mice. **(A)** Heatmap of k-means clustering showing DEGs in hepatic endothelial cells between lean and obese mice at 0, 7, and 28 d.p.i. **(B, C)** GO enrichment analysis of endothelial DEGs between lean and obese at 7 **(B)** and 28 **(C)** d.p.i. **(D)** Violin plots showing gene signature scores associated with positive regulation of platelet aggregation and prothrombotic responses in endothelial cells across the six groups.

These differences persisted during recovery. At 28 d.p.i., endothelial cells from lean mice were associated with vasculature development, blood vessel morphogenesis, angiogenesis, cell junction assembly, and endothelial cell migration. However, endothelial cells from obese mice showed upregulation of pathways linked to the unfolded protein response, humoral immune response, B cell differentiation, and antigen processing and presentation, features suggestive of prolonged inflammatory signaling and immune engagement (**Figure 4C**). Additionally, endothelial cells from obese mice had an increase in positive regulation of platelet aggregation and prothrombotic pathways (**Figure 4D**), which may contribute to an elevated risk of liver fibrosis and vascular complications (25). Hepatic endothelial cells on obese mice together retain an immune-activated and prothrombotic transcriptional state during the post-acute phase rather than acquiring the vascular homeostatic signatures observed in lean mice.

### Hepatic macrophages retain inflammatory and metabolic alterations in obese mice during recovery​

Since dysregulated macrophage activation can contribute to sustained inflammation and tissue injury during viral infection (18, 20, 21, 26, 27) and the prominent myeloid response during acute infection, we next resolved liver monocyte and macrophage heterogeneity. Re-clustering identified recruiting monocytes (rMon), monocyte-derived macrophages (MDM), intermediate monocyte-derived macrophages (interMoM), inflammatory MDM (infMDM), M2-like macrophages, Kupffer cells, lipid-associated macrophages (LAMs), plasmacytoid dendritic cells (pDCs), conventional DCs, and migratory DCs (**Figures 5A, B**). In obese mice, rMon decreased at day 7, whereas MDMs increased during the post-acute phase and were slightly higher than in lean mice at day 28. M2-like macrophages were higher in obese mice at baseline and remained relatively abundant following an infection, although they declined over time. Interestingly, LAMs and infMDMs were consistently increased in obese mice at both 7 and 28 d.p.i. (**Figure 5C**). These differences suggest that obesity is associated with sustained remodeling of the liver macrophage compartment, which may contribute to worsened pathology following an infection.

**Figure 5:**
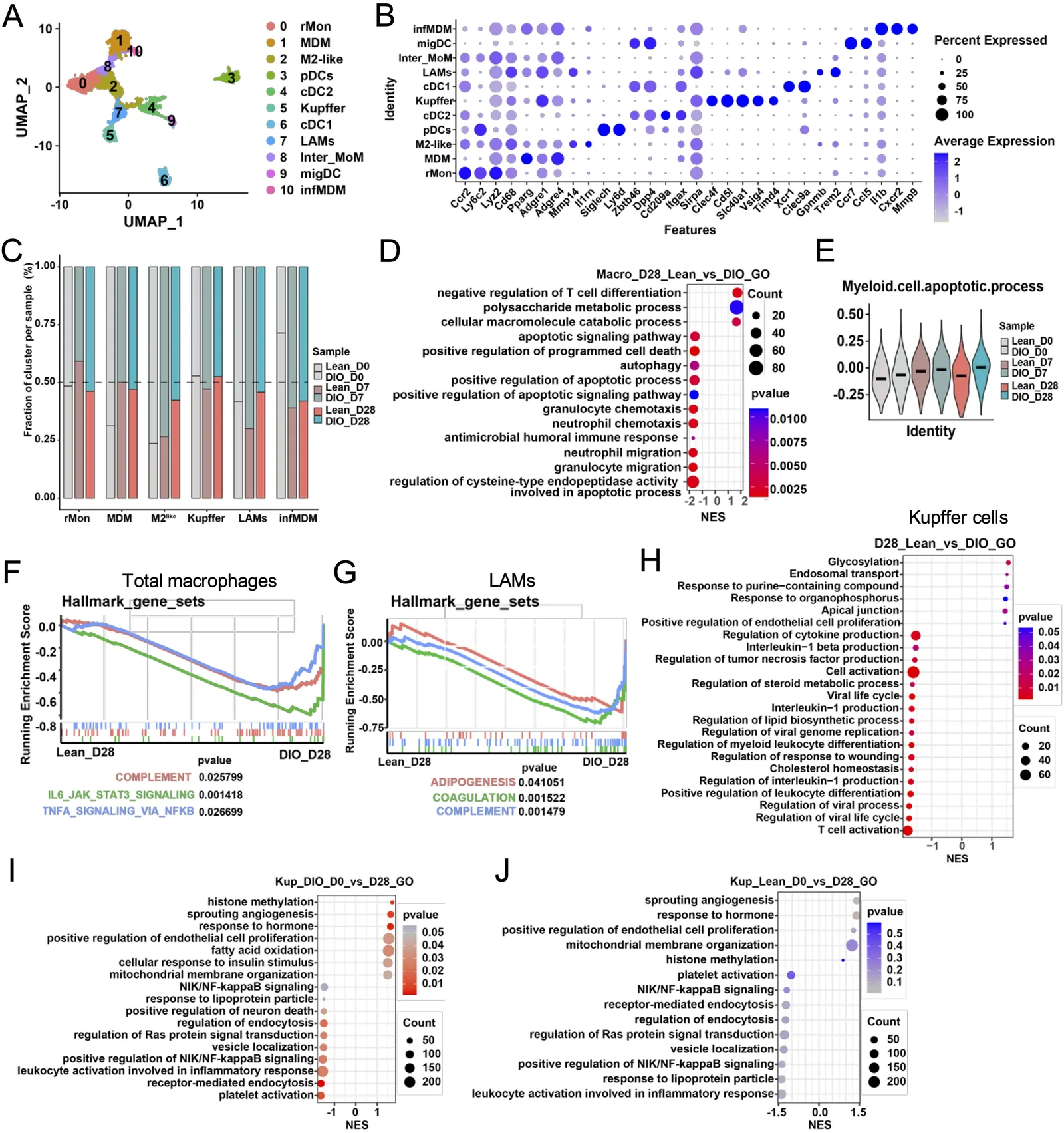
Hepatic macrophages retain inflammatory and metabolic alterations in obese mice during recovery. **(A)** UMAP projection of reclustered hepatic myeloid populations. **(B)** Dot plot showing the expression of typical marker genes for myeloid cell types. **(C)** Relative contributions of selected monocyte/macrophage clusters across lean and obese mice at 0, 7, and 28 d.p.i. **(D)** GO enrichment analysis comparing the total hepatic macrophages between lean and obese mice at 28 d.p.i. **(E)** Violin plots showing apoptotic-process gene signature scores in myeloid cell among experimental conditions. **(F, G)** GSEA of hallmark pathways in total hepatic macrophages **(F)** or LAMs **(G)** comparing lean and obese mice at 28 d.p.i. **(H–J)** GO enrichment analysis of Kupffer cells comparing lean and DIO mice at 28 d.p.i. **(H)** DIO mice at 0 and 28 d.p.i. **(I)** and lean mice at 0 and 28 d.p.i. **(J)**.

We then examined whether these changes in macrophage composition were linked to persistent transcriptional alterations. At 28 d.p.i., overall, macrophages from obese mice remained transcriptionally distinct from those of lean mice, with enrichment of multiple immune and stress-associated processes, including apoptotic signaling, programmed cell death, autophagy, granulocyte/neutrophil chemotaxis, antimicrobial humoral response, and cysteine-type endopeptidase activity (**Figures 5D, E**). Consistent with this inflammatory phenotype, the GSEA of total macrophages showed enrichment of complement, IL6-JAK-STAT3, and TNF-NF-κB signaling in obese mice (**Figure 5F**). Interestingly, LAMs showed a distinct but related transcriptional signature, characterized by enrichment of adipogenesis, coagulation, and complement pathways in obese mice (**Figure 5G**). In contrast to several recruited macrophage populations, the abundance of Kupffer cells remained largely comparable between lean and obese mice. However, Kupffer cells from obese mice upregulated pathways associated with metabolic processes, IL-1 production, and T cell activation compared to lean mice (**Figure 5H**). Moreover, a comparison of obese mice at 28 d.p.i. with their baseline controls revealed reduced fatty acid oxidation and cellular response to insulin, along with increased leukocyte activation and NIK/NF-κB signaling (**Figure 5I**), whereas these infection-associated changes were less pronounced in lean mice (**Figure 5J**). These findings altogether highlight persistent inflammatory and metabolic remodeling of hepatic macrophages in obese mice during the post-acute phase, suggesting that altered macrophage state may contribute to impaired resolution of hepatic inflammation and metabolic homeostasis following infection.

### Obesity alters hepatic T cell transcriptional profiles during SARS-CoV-2 infection

Given the changes in liver lymphocyte populations following infection, we next examined the transcriptional response of T cells. Sub-clustering identified distinct T cell subsets based on established markers (**Figures 6A, B**), which exhibited substantial changes across disease stages (**Figure 6C**). At 7 d.p.i., T cells from obese livers exhibited broad enrichment of metabolic and proliferative programs, including oxidative phosphorylation, glycolysis, E2F and Myc targets, and G2/M checkpoint pathways, together with enhanced mTORC1, IL-2/STAT5, and interferon-gamma response signatures (**Figure 6D**). In contract, Wnt/β-catenin signaling was reduced in obese mice. These results indicate that obesity is associated with a distinct metabolically active and inflammatory T cell state during acute infection, suggesting that altered T cell response may involve in the heightened immune environment in obese livers.

**Figure 6:**
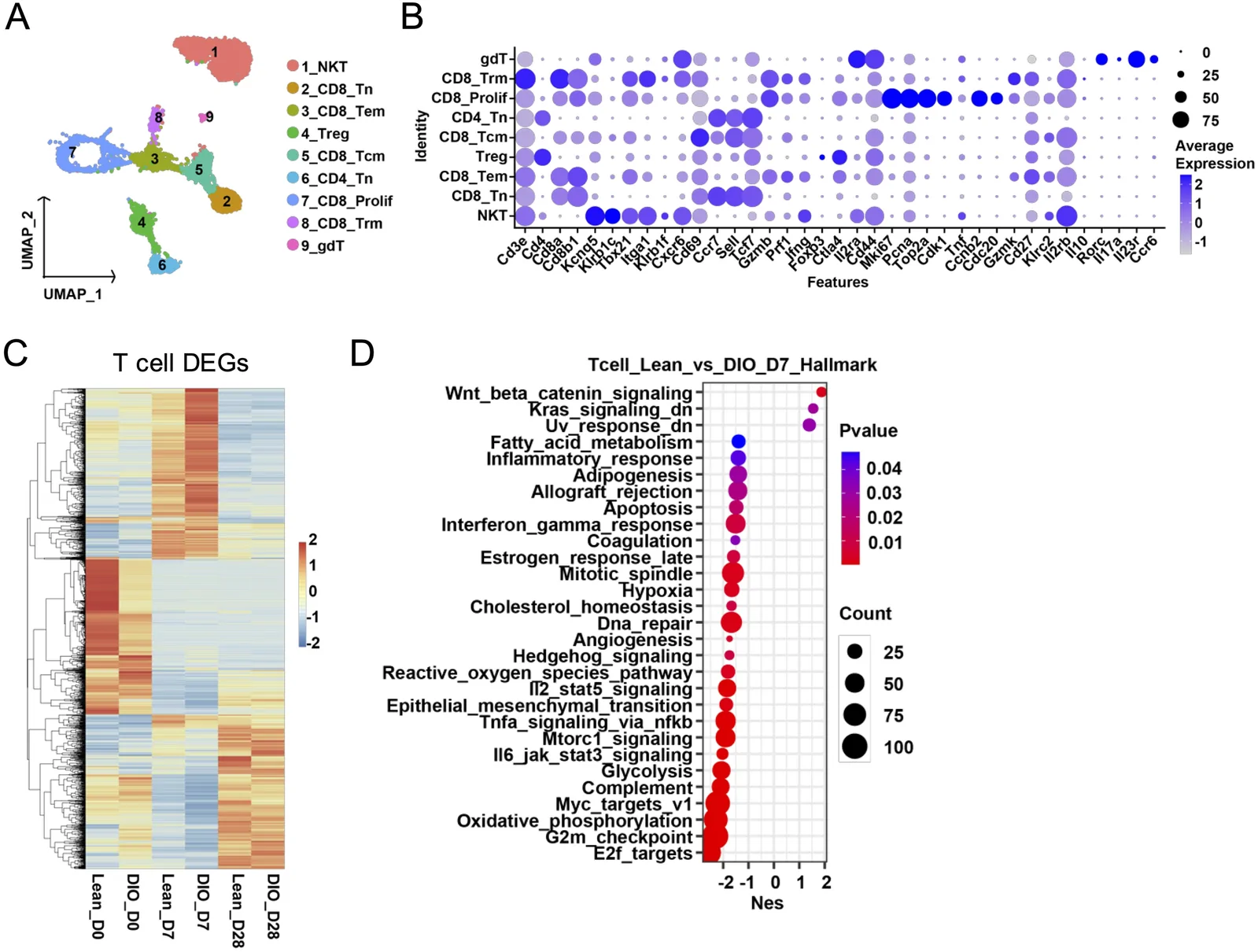
Obesity alters hepatic T cell transcriptional profiles during SARS-CoV-2 infection. **(A)** UMAP projection of reclustered T cell subpopulations. **(B)** Dot plot showing the representative marker gene expression used to annotate T cell subsets. **(C)** Heatmap of k-means showing DEGs in hepatic T cells across the six study groups. **(D)** Hallmark pathway enrichment analysis of T cell DEGs between lean and obese mice at 7 d.p.i.

### Obese mice exhibit persistent hepatocyte metabolic remodeling during recovery

Hepatocytes are the primary cell types in the liver and play a pivotal role in liver metabolism and function. Given the presence of hepatocyte death in infected obese mice, we analyzed the transcriptional profile of hepatocytes following infection. We focused on pathways which are commonly disrupted during liver fibrosis (28). Hepatocytes from obese mice displayed higher signatures for gluconeogenesis and fatty acid uptake than those from lean mice, with the greatest uptake observed at 28 d.p.i. (**Figure 7A**). At 7 d.p.i., when compared to lean mice, both hepatocytes and Kupffer cells from obese mice appeared to have upregulated lipogenesis-related and downregulated lipolysis-related signatures (**Figure 7B**), consistent with higher lipid droplet accumulation in obese liver at this time point. In addition, hepatocytes from obese mice showed an increase in apoptosis-associated programs at day 7, followed by an increase in adipogenesis-associated signatures at day 28 (**Figures 7C, D**). Consistent with these changes, pathway analysis at 7 d.p.i. revealed enrichment of cholesterol homeostasis, glycolysis, peroxisome, adipogenesis, and fatty acid metabolism in obese compared with lean hepatocytes (**Figure 7E**). By day 28, lean hepatocytes exhibited upregulation of pathways involved in lipopolysaccharide response, peptide and amide transport, bacterial defense, and wound healing, whereas obese hepatocytes showed enrichment of pathways associated with leukocyte-mediated cytotoxicity, collagen biosynthesis, mesenchymal cell proliferation, T cell receptor signaling, and natural killer cell-mediated cytotoxicity (**Figure 7F**), indicating ongoing inflammation and fibrotic remodeling in obese liver.

**Figure 7:**
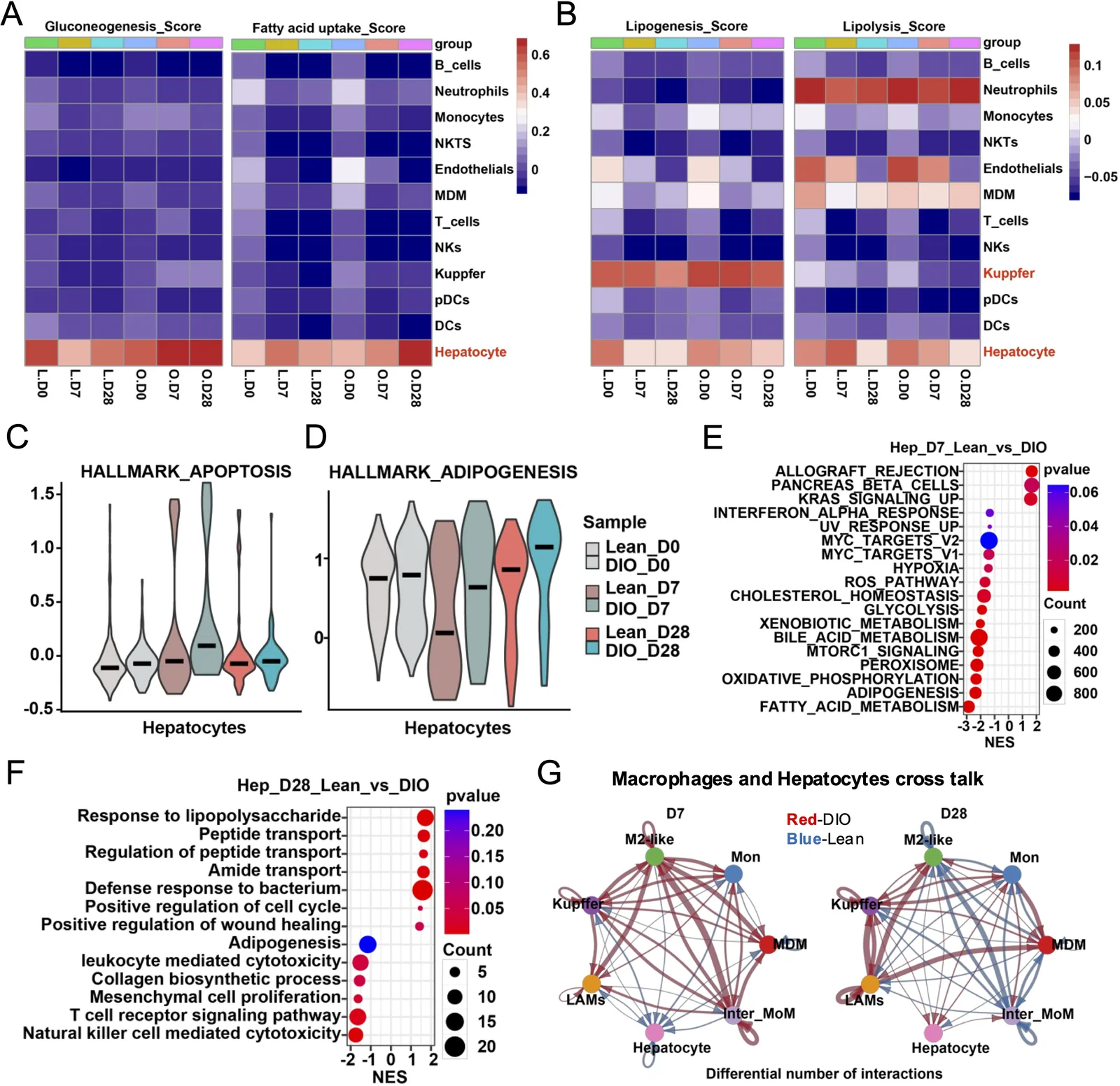
Obese mice exhibit persistent hepatocyte metabolic remodeling during recovery. **(A, B)** Heatmaps showing gluconeogenesis, fatty acid uptake, lipogenesis, and lipolysis signature scores across all of the cell populations and time points. **(C, D)** Violin plots showing apoptosis-associated **(C)** and adipogenesis-associated gene signature scores in hepatocytes across the six conditions. **(E, F)** Pathway enrichment analysis of hepatocyte comparing lean and DIO mice at 7 d.p.i. **(E)** and 28 d.p.i. **(F)**. **(G)** CellChat analysis comparing predicted ligand–receptor interaction networks among hepatocytes and macrophage clusters at 7 and 28 d.p.i. The red and blue edges indicate interactions relatively enriched in DIO and lean mice, respectively, and edge thickness represents the relative strength of predicted interactions.

Both macrophages and hepatocytes retained transcriptional alterations during recovery. We next used CellChat to explore potential changes in intercellular communication between these populations. Predicted interaction networks differed between lean and obese mice and evolved over the course of infection (**Figure 7G**). At 7 d.p.i., obese mice displayed broader communication among macrophages, whereas hepatocytes in lean mice received more incoming signals. This pattern was shifted at day 28, with increased incoming communications to hepatocytes in obese mice and enhanced interactions between LAMs and Kupffer cells, MDMs, or M2-like macrophages (**Figure 7G**). These analyses together suggest that persistent hepatocyte metabolic remodeling in obese mice is associated with altered macrophage–hepatocyte communication during post-acute infection, providing a potential link between sustained immune dysregulation and impaired metabolic recovery.

## Discussion

Our study reveals that obesity alters the trajectory of hepatic responses following SARS-CoV-2 infection. Obese mice developed greater hepatic metabolic abnormalities, hepatocellular apoptosis, and myeloid inflammation during acute infection. Although several features of acute injury and inflammation diminished during recovery, the obese liver remained transcriptionally distinct at 28 d.pi., with persistent alterations across immune, endothelial, and parenchymal compartments. These findings indicate that the impact of obesity extends beyond exacerbating acute disease and is associated with incomplete restoration of hepatic homeostasis during the post-acute phase.

A major finding of this study is that obesity is associated with prolonged tissue pathology following SARS-CoV-2 infection. Obese mice exhibited persistent pulmonary inflammation and fibrosis together with evidence of liver injury at 28 d.p.i., whereas these pathological features were less prominent in lean mice during recovery. These observations support emerging clinical evidence that obesity is a key risk factor not only for severe acute COVID-19 but also for the development of long-term complications (29, 30). More broadly, the persistence of tissue abnormalities in obese mice suggests that pre-existing metabolic dysfunction may limit the restoration of tissue homeostasis following acute viral infection.

The mechanisms underlying liver injury following viral infection in the setting of obesity remain incompletely understood (31). Our histological analyses revealed increased lipid accumulation, hepatocellular apoptosis, and liver dysfunction in infected obese mice. These findings are consistent with the concept that lipid accumulation and metabolic stress in steatotic hepatocytes can increase their susceptibility to inflammatory injury and amplify local immune response (32). SARS-CoV-2 infection may therefore act as an additional stressor that exacerbates pre-existing metabolic abnormalities, accelerating hepatocellular injury and impairing recovery. On the other hand, immune dysregulation from obesity may intensify viral pathology (16, 33), while viruses exploit lipid pathways to enhance replication (34, 35). Notably, the increased lipid deposition observed during post-acute infection suggests that viral-induced metabolic remodeling persists after the acute inflammatory phase and may contribute to progressive liver dysfunction (36). Given the high prevalence of metabolic dysfunction-associated steatotic liver disease (MASLD) worldwide, these findings have important implications for understanding the long-term hepatic consequences of COVID-19 in vulnerable populations.

Single-cell transcriptomic analysis provided a cellular framework for understanding the persistent hepatic alterations observed after infection. We identified sustained remodeling of the hepatic macrophage compartment in obese mice, including increased recruited macrophage populations and persistent inflammatory transcriptional programs during recovery. Macrophages are central regulators of both tissue injury and repair, and dysregulated macrophage responses have been implicated in chronic inflammatory diseases and fibrosis (26, 37, 38). In the context of SARS-CoV-2 infection, macrophage inflammatory states can further shape tissue-cell responses and injury (39). Our findings suggest that obesity is associated with persistent inflammatory macrophage states during recovery. Such states may perpetuate cytokine production, amplify hepatocellular stress, and disrupt tissue repair pathways, thereby contributing to chronic liver injury following SARS-CoV-2 infection. Notably, persistent alterations were not restricted to macrophages. At 28 d.p.i., hepatic endothelial cells from obese mice exhibited increased prothrombotic and platelet aggregation-associated transcriptional signatures, consistent with the vascular and thromboinflammatory abnormalities reported in obese patients with COVID-19 (40, 41). Hepatocytes also exhibited substantial metabolic remodeling, including enhanced gluconeogenic signatures and widespread perturbations in lipid metabolic programs. Hepatocytes play a central role in maintaining systemic metabolic homeostasis, and dysregulation of glucose and lipid metabolism can profoundly influence inflammatory responses (42, 43). Persistent alterations in these programs may therefore reflect impaired hepatic metabolic regulation during recovery. These findings together indicate that post-acute abnormalities in obese livers extend across immune, vascular, and parenchymal compartments, potentially contributing to impaired hepatic recovery following infection.

Importantly, our study highlights the close interplay between immune and metabolic pathways during post-acute hepatic recovery. Chronic inflammation and metabolic dysfunction are interconnected in obesity, and persistent inflammatory signaling can influence hepatocyte metabolism, while metabolic stress within hepatocytes may reciprocally shape local immune responses (43–45). Consistent with this concept, the coordinated alterations observed among macrophage and hepatocyte compartments, together with dynamic changes in predicted intercellular communications, suggest that disruption of immunometabolic interactions may contribute to impaired hepatic recovery after SARS-CoV-2 infection. Effective recovery from viral injury requires the coordinated resolution of inflammation and activation of tissue repair and regenerative programs (26, 46, 47). Our findings suggest that these processes remain altered in obese livers during recovery, resulting in a hepatic state characterized by persistent inflammatory and metabolic remodeling. Thus, incomplete recovery of hepatic immunometabolic homeostasis may represent one mechanism through which pre-existing metabolic dysfunction influences post-acute tissue outcomes following SARS-CoV-2 infection. Future studies will be important to determine whether persistent macrophage–hepatocyte interactions actively sustain these post-acute metabolic changes and whether targeting specific immunometabolic pathways can promote hepatic recovery.

Several limitations should be considered. The MA10 mouse model does not capture the full complexity of human PASC, and direct validation in human liver samples will be important. In addition, scRNA-Seq was performed using pooled liver samples for each condition, limiting biological replication of the transcriptomic comparisons. Pathway enrichment and CellChat analyses are based on computational predictions and, therefore, identify candidate cellular processes and interactions rather than establishing metabolic function or causal mechanisms. Finally, the 28-day endpoint captures an early post-acute period, and longer-term studies will be required to determine the persistence and functional consequences of these alterations.

In conclusion, our study demonstrates that obesity is associated with persistent hepatic immunometabolic remodeling following SARS-CoV-2 infection. Single-cell transcriptomic profiling identified inflammatory macrophage populations and altered hepatocyte metabolic programs as key features of obesity-associated liver sequelae, providing a cellular framework for understanding incomplete hepatic recovery in metabolically compromised hosts. These findings highlight the restoration of tissue immunometabolic homeostasis as an important dimension of recovery from viral infection and identify candidate cellular pathways for future mechanistic investigation.

## Data Availability

The scRNA-seq data generated in this study was deposited in GEO under accession number GSE308734. Any additional information required to reanalyze the data reported in this paper is available from the lead contact upon request.

## Supplementary material

Document S1 **Supplementary Figures S1**-**S3**.

Supplementary Table 1 List of antibodies and reagents used in study.
