# Supplementary material for: Single-cell transcriptomics uncovers persistent hepatic immunometabolic remodeling following SARS-CoV-2 infection in obese hosts
Source: Front Immunol. 2026 Oct 14;17:1910050. doi: 10.3389/fimmu.2026.1910050 (PMC13713652; doi:10.3389/fimmu.2026.1910050)
Supplement: Supplementary file 1 [file Table1.docx]

**KEY RESOURCES TABLE**

| REAGENT or RESOURCE | SOURCE | IDENTIFIER |
| --- | --- | --- |
| **Antibodies** | | |
| PerCP/Cyanine5.5 anti-mouse CD45 Antibody | BioLegend | Cat# 103132; RRID AB_893340 |
| Brilliant Violet 510™ anti-mouse CD11c Antibody | BioLegend | Cat# 117338; RRID AB_2562016 |
| FITC anti-mouse/human CD11b Antibody | BioLegend | Cat# 101206; RRID AB_312789 |
| PE/Cyanine7 anti-mouse Ly-6G Antibody | BioLegend | Cat# 127618; RRID AB_1877261 |
| Brilliant Violet 711™ anti-mouse Ly-6C Antibody | BioLegend | Cat# 128037; RRID AB_2562630 |
| Brilliant Violet 605™ anti-mouse I-A/I-E Antibody | BioLegend | Cat# 107639; RRID AB_2565894 |
| PE anti-mouse F4/80 Antibody | BioLegend | Cat# 123110; RRID AB_893486 |
| Brilliant Violet 785™ anti-mouse CD4 Antibody | BioLegend | Cat# 100552; RRID AB_2563053 |
| Brilliant Violet 421™ anti-mouse CD8a Antibody | BioLegend | Cat# 100753; RRID AB_2562558 |
| Brilliant Violet 510™ anti-mouse/human CD44 Antibody | BioLegend | Cat# 103044; RRID AB_2650923 |
| Brilliant Violet 650™ anti-mouse CD19 Antibody | BioLegend | Cat# 115541; RRID AB_11204087 |
| PE/Cyanine7 anti-mouse NK-1.1 Antibody | BioLegend | Cat# 156513; RRID AB_2888852 |
| PE anti-Spike_539-546_ | NIH Tetramer Core Facility | N/A |
| Anti-Collagen I antibody | Abcam | Cat# ab270993 |
| Anti-alpha smooth muscle Actin antibody [1A4] | Abcam | Cat# ab7817 |
| Goat anti-Rabbit IgG (H+L) Cross-Adsorbed Secondary Antibody, Alexa Fluor™ 488 | Invitrogen | Cat# A-11008; RRID AB_143165 |
| Goat anti-Mouse IgG (H+L) Cross-Adsorbed Secondary Antibody, Alexa Fluor™ 555 | Invitrogen | Cat# A-21422; RRID AB_2535844 |
| **Bacterial and virus strains** | | |
| SARS-CoV-2, MA10 strain | This paper | N/A |
| **Chemicals, peptides, and recombinant proteins** | | |
| Dako Target Retrieval Solution, pH 9 | Agilent | Cat# S236784-2 |
| Dako Antibody Diluent | Agilent | Cat# S302283-2 |
| Tween-20 | Sigma | Cat# P7949 |
| DAPI | Thermo Scientific | Cat# 62248 |
| ProLong™ Gold Antifade Mountant | Invitrogen | Cat# P36930 |
| Xylenes | Sigma | Cat# 534056 |
| Triton X-100 | Fisher Scientific | Cat# M1122980101 |
| 10% Neutral Buffered Formalin | Fisher Scientific | Cat# 22-110-869 |
| Fc Receptors Blocker | Innovex | Cat# NB309 |
| Fetal Bovine Serum | Cytiva | Cat# SH30070.03 |
| Zombie NIR™ Fixable Viability Kit | BioLegend | Cat# 423106 |
| RBC Lysis Buffer (10X) | BioLegend | Cat# 420302 |
| Hanks' Balanced Salt Solution (HBSS) | Gibco | Cat# 14175095 |
| fatty acid free BSA | Thermo Scientific | Cat# J64944.30 |
| collagenase IV | Sigma | Cat# C4-22-1G |
| Percoll | Sigma | Cat# P1644 |
| ETGA | Boston-bioproducts | Cat# BM-151 |
| **Critical commercial assays** | | |
| Chromium Next GEM Single Cell 3ʹ Reagent Kits v3.1 (Dual Index) | 10x Genomics | CG000315 Rev D |
| TUNEL Assay Kit - HRP-DAB | Abcam | Cat# ab206386 |
| Glucose Assay Kit | Abcam | Cat# ab65333 |
| Cholesterol Assay Kit | Abcam | Cat# ab65390 |
| Mouse ALT ELISA Kit | Abcam | Cat# ab282882 |
| **Deposited data** | | |
| Raw data scRNA-Seq | This paper |  |
|  |  |  |
| **Experimental models: Cell lines** | | |
| Vero E6 cells | This paper | N/A |
| **Experimental models: Organisms/strains** | | |
| C57BL/6J DIO | Jackson Laboratory | Strain # 380050 |
| C57BL/6J DIO Control | Jackson Laboratory | Strain # 380056 |
| **Software and algorithms** | | |
| RStudio | R | N/A |
| ZEN blue 3.7 | Zeiss | N/A |
| OlyVIA | Olympus | N/A |
| QuPath (v0.4.3) | QuPath | N/A |
| FlowJo (v10.8.1) | BD Biosciences | N/A |
| BioRender | BioRender | N/A |
| Graphpad Prism 9 | Graphpad | N/A |
| **Other** | | |
| Ketamine, 100MG/ML | Covetrus | Cat# 010177 |
| Xylazine, 100MG/ML | Covetrus | Cat# 061035 |
| Rodent Diet with 60% kcal% fat | Research Diets | Cat# D12492 |
